# Development of estimates of dietary nitrates, nitrites, and nitrosamines for use with the short willet food frequency questionnaire

**DOI:** 10.1186/1475-2891-8-16

**Published:** 2009-04-06

**Authors:** John S Griesenbeck, Michelle D Steck, John C Huber, Joseph R Sharkey, Antonio A Rene, Jean D Brender

**Affiliations:** 1 Department of Social and Behavioral Health, School of Rural Public Health, Texas A&M Health Science Center, College Station, TX, USA

## Abstract

**Background:**

Studies have suggested that nitrates, nitrites, and nitrosamines have an etiologic role in adverse pregnancy outcomes and chronic diseases such as cancer. Although an extensive body of literature exists on estimates of these compounds in foods, the extant data varies in quality, quantified estimates, and relevance.

**Methods:**

We developed estimates of nitrates, nitrites, and nitrosamines for food items listed in the Short Willet Food Frequency Questionnaire (WFFQ) as adapted for use in the National Birth Defects Prevention Study. Multiple reference databases were searched for published literature reflecting nitrate, nitrite, and nitrosamine values in foods. Relevant published literature was reviewed; only publications reporting results for items listed on the WFFQ were selected for inclusion. The references selected were prioritized according to relevance to the U.S. population.

**Results:**

Based on our estimates, vegetable products contain the highest levels of nitrate, contributing as much as 189 mg/serving. Meat and bean products contain the highest levels of nitrites with values up to 1.84 mg/serving. Alcohol, meat and dairy products contain the highest values of nitrosamines with a maximum value of 0.531 μg/serving. The estimates of dietary nitrates, nitrites, and nitrosamines generated in this study are based on the published values currently available.

**Conclusion:**

To our knowledge, these are the only estimates specifically designed for use with the adapted WFFQ and generated to represent food items available to the U.S. population. The estimates provided may be useful in other research studies, specifically in those exploring the relation between exposure to these compounds in foods and adverse health outcomes.

## Background

Studies have suggested that nitrates, nitrites, and nitrosamines have an etiologic role in adverse pregnancy outcomes and other health conditions [1-5]. Nitrates and nitrites are precursors in the formation of *N*-nitroso compounds, a class of genotoxic compounds consisting of nitrosamines and nitrosamides [2]. *N*-nitroso compounds are known to cause congenital malformations in animal models, and the role of these compounds in adverse pregnancy outcomes warrants further exploration [6-8]. Humans are exposed to nitrates primarily through diet and drinking water, with vegetables contributing the largest amount of dietary nitrates per serving [2,9,10]. Nitrates are inherently present in all plant materials, especially vegetables and forage crops, and accumulate when the plant matures in a nitrate rich environment [11]. Nitrates in drinking water are often the result of contamination of ground water by fertilizer and animal or human waste [2]. The interest in nitrate consumption is due to the subsequent conversion of nitrates to nitrites, which are of greater concern in the formation of *N*-nitroso compounds. The endogenous conversion of nitrate to nitrite is a significant source of exposure to nitrites; approximately 5% of ingested nitrates in food and water are converted to nitrite in the saliva [12]. Cured meats, baked goods and cereals are other notable sources of nitrite [13]. Nitrite salts are added to meats, poultry, and fish in minute quantities as a means of preservation; this has been a common practice for many centuries [14]. Humans are exposed to *N*-nitroso compounds from exogenous sources and through endogenous formation. Dietary sources of nitrosamines include cured meats, beer, and smoked fish; these foods may contain preformed nitrosamines as the result of cooking and/or preservation methods [14-16]. Non-dietary sources include tobacco products, cosmetics, and occupational exposures in rubber or rocket fuel factories and leather tanneries [17-21].

As part of an ongoing study of the relation between prenatal exposure to these compounds and selected congenital malformations in offspring, we developed estimates of nitrates, nitrites, and nitrosamines for food items in the Short Willet Food Frequency Questionnaire (WFFQ) [22,23] as adapted for the National Birth Defects Prevention Study (NBDPS) [24]. Although an extensive body of literature exists on estimates of these compounds in foods, the extant data varies in quality, quantified estimates, country of origin and relevance to the U.S. population. Much of the literature was published before 1970 and may not accurately reflect current technological advances in laboratory methods and food preservation techniques. Furthermore, published estimates of nitrates, nitrites, and nitrosamines are not available for the WFFQ, a food frequency questionnaire that is commonly used in research studies.

## Methods

The NBDPS study collects dietary information using the adapted WFFQ [22,23]. This adaptation was designed to make the questionnaire more suitable to the ethnic and racially diverse NBDPS study population and includes additional food items such as avocados, raw chili peppers, salsa, tortillas, cantaloupe, and refried beans.

Four steps were required to generate estimates of nitrates, nitrites, and nitrosamines for food items in the WFFQ: data collection, creation of a food database, selecting relevant values from that food database for our study population, and finally generating a summary estimate for each food item. To generate estimates, it was necessary to first develop a food database containing all relevant information associated with each published value (mean, range, cooking method, country, author, year, etc.) obtained from our literature search. This food database was consulted to identify appropriate values for our population based on our ranking system which accounted for the year(s) of sample collection and country of origin. Finally, for each food item we calculated a summary estimate based on the relevant values selected from the food database.

### Step 1: Data Collection

A thorough literature review was conducted to identify and categorize relevant reported values of nitrate, nitrite, and nitrosamine in foods. Multiple reference databases were searched for published literature reflecting nitrate, nitrite, and nitrosamine values in foods and alcoholic beverages. Reference databases queried included Medline, ISI Web of Science, Agricola, and Google Scholar. Each reference database was searched using the following terms: "nitrates," "nitrites," "nitrosamines," "food content," "dietary sources," and combinations of the listed terms. Relevant published literature was reviewed but only sources reporting results for the food items listed on the WFFQ as adapted for the NBDPS were selected for inclusion [22-24]. For alcoholic beverages (beer, wine, liquor, and malt beverages) and for food items that returned no values for nitrates, nitrites, or nitrosamines an additional literature review was conducted. Food item estimates that were not available in the literature were substituted with similar food items with reported values. For example, a nitrosamine value for "orange juice" was not located using the search criteria previously mentioned; therefore, the value for "oranges" was used as a comparable substitution. There were also situations where some of the missing food items required recipes to generate an appropriate estimate. For example, a nitrate value for "salsa" was calculated using values for "onions", "peppers", and "tomatoes" in the proportions designated in a commonly available cookbook [25].

### Step 2: Database Creation

The food database was created as part of the process to generate summary estimates that are used to determine dietary intake of these compounds for U.S. women participants (giving birth during 1997 – 2005) in the NBDPS. Although the study period encompasses the years 1997–2005; we were not able to limit our estimates to values published during this time period. Because of the lack of current values in the literature, it was necessary to expand our search criteria to include values from other countries and published during other time periods. The identified values were prioritized within each food item according to country of origin and the year of sample collection. The year of sample collection was used in order to select the time period that most accurately reflected the values of these compounds in foods consumed by the NBDPS study population. However, the sample year was not always available; in those situations the year the article was received by the journal was used. When the above information was unavailable, we defaulted to the year of publication. Studies have shown that nitrate, nitrite, and nitrosamine content in food products likely varies over time and by region [26,27]. To develop the most accurate and relevant values in terms of time and place for the NBDPS study population, priority was given to estimates from Western countries from 1980 to present. The ranks ranged from one (highest) to five (lowest) and are as follows: 1) U.S. and Canada 1980 to present; 2) countries with predominantly Western diets 1980 to present; 3) U.S. and Canada 1970–1979; 4) countries with predominantly Western diets 1970–1979; and 5) countries with predominantly non-Western diets. Thirty-four articles and four government reports were selected for inclusion in the food database and are listed in Table 1. As available, information was abstracted regarding cooking or preservation methods, methods of laboratory analysis, number of observations in each sample, ranges of values, means, and standard deviations. Although not specifically published in this article, the food database is available upon request.

**Table 1:** References used in food database and estimate generation

Citation	Year(s) Values Collected^a^	Country^b^	Rank^c^	Included in Estimate^d^
Canas, et al., 1986 [36]	1985	US	1	√
Chung, et al., 2003 [37]	1998	KR	5	√
Cornee, et al., 1992 [35]	1992	FR	2	√
Dennis, et al., 1984 [38]	1983	IS	5	-
Food Standards Agency, 1998 [39]	1997	UK	2	√
French National Inventory, 1982 [40]	1982	FR	2	√
Fudge and Truman, 1973 [41]	1973	UK	4	-
Havery, et al., 1982 [42]	1981	US	1	√
Howe, et al., 1986 [43]	1985	CA	1	√
Huang, et al., 1981 [44]	1980	HK	5	-
Key, et al., 1982 [45]	1982	UK	2	√
Klein, et al., 1980 [46]	1980	FR	2	√
Knight, et al., 1987 [47]	1973–1978	UK	4	-
Kyriakidis, et al., 1997 [48]	1995	GR	5	-
Lakritz and Pensabene, 1981 [49]	1980	US	1	√
Libbey, et al., 1980 [50]	1979	US	3	√
Mahieu, et al., 1980 [51]	1980	FR	2	√
Maki, et al., 1980 [52]	1976–1980	JP	5	√
Matsui, et al., 1980 [53]	1979	JP	5	-
Merino, et al., 2006 [54]	1995–2005	SE	2	√
National Academy of Sciences, 1981a [13]	1975–1979	US	3	√
Osterdahl, 1988 [55]	1980–1986	SE	2	√
Pedersen and Meyland, 1981 [56]	1981	DE	2	√
Petersen and Stoltze, 1999 [57]	1993–1997	DK	2	√
Pobel, et al., 1995 [58]	1994	FR	2	√
Saccani and Tanzi, 2006 [59]	2005	IT	2	√
Scanlan, et al., 1980 [60]	1979	US	3	-
Sen, et al., 1980 [61]	1979	CA	3	√
Sen, et al., 1988 [62]	1988	CA	1	√
Siciliano, et al., 1975 [63]	1973–1974	US	3	√
Siddiqi, et al., 1988 [64]	1988	IN	5	-
Spiegelhalder, et al., 1980 [65]	1980	DE	2	√
Tamme, et al., 2006 [66]	2003–2004	EE	5	√
Thomson and Swallow, 2004 [67]	2003	NZ	2	√
Tricker, et al., 1991 [68]	1989–1990	DE	2	√
Vecchio, et al., 1986 [69]	1984	US	1	√
VonCollett, 1983 [70]	1983	DE	2	√
Yamamoto, et al., 1984 [71]	1982	JP	5	√

^a^Year(s) sample collected is reported, when not available year received by journal is used and year of publication is used as when all other dates are unavailable

^b^CA = Canada, DE = Germany, DK = Denmark, EE = Estonia, FR = France, GR = Greece, HK = Hong Kong, IN = India, IS = Iceland, IT = Italy, JP = Japan, KR = South Korea, NZ = New Zealand, SE = Sweden, UK = United Kingdom, US = United States

^c^Rank 1 = U.S. and Canada 1980 to present; Rank 2 = countries with predominantly Western diets 1980 to present; Rank 3 = U.S. and Canada 1970–1979; Rank 4 = countries with predominantly Western diets 1970–1979; and Rank 5 = countries with predominantly non-Western diets

^d^(√) = included in estimate; (-) = excluded from estimate

### Step 3: Value Selection

Values compiled in the food database from the United States and Canada (rank 1) and other countries with traditionally Western diets (rank 2) from 1980 to present were automatically used to calculate the estimates for nitrates, nitrites, and nitrosamines in each food item. When there were fewer than five values reported for a food item ranked 1 or 2, values from the United States and Canada from 1970–1979 were used. Less relevant values with respect to country and year were used when no other estimates were available.

### Step 4: Estimate Generation

Table 1 lists the 30 references that met the value selection criteria for generating summary estimates of nitrates, nitrites, and nitrosamines in food items. A summary estimate for each food item was generated through a multi-step process using the mean or midpoint and the sample sizes from each study that met our selection criteria. A systematic approach to calculate these estimates was developed using STATA 10 [28]. A summary estimate for each food item was calculated as the weighted mean of the published values for that food item identified from the food database. The weights were calculated as the square root of the reported sample size for each value used to formulate the summary estimate. We would have preferred to use the inverse of the variance to weight the values but many of the reported values did not include a variance estimate. This process was implemented to create weighted total estimates that were representative of the reported values. The estimates are based on reported values selected to represent the U.S. food supply and potentially represent the exposure of the U.S. population to these compounds. This procedure was repeated for each food item.

The WFFQ as adapted for the NBDPS asks about dietary intake of 64 food items, some of which are categories of foods; however, we developed a single summary estimate per compound (nitrate, nitrite, and nitrosamines) for each food item. The process was straightforward in the case of individual food items, but required an additional procedure for category items. For example, the "processed meats" item contains component estimates for sausage, salami, lunchmeat, pâté, and preserved meat. To generate a single estimate for "processed meats", weighted estimates for each individual component of that group were calculated. The summary estimate for "processed meats" was calculated by weighting the component estimates equally. The summary estimates were reported in mg/100 g for nitrates and nitrites and μg/100 g for nitrosamines.

For each food item, nitrates, nitrites, and nitrosamines were calculated for a typical serving using the summary estimates and the standard serving size in grams as defined by the NBDPS Nutrient Calculator when available [29]. For cereal, biscuits, fruit drinks, soy milk and tofu, the standard serving sizes were obtained using nutrition information provided on the label of commonly available products from a local grocery store.

The NBDPS Mother Questionnaire collects information regarding intake of alcoholic beverages by frequency and type of alcohol consumed. The questionnaire divides alcoholic beverages into five broad categories identified as beer, wine, mixed drinks, shot liquor, and other alcohol. We used standard serving sizes of alcohol in the United States as reported by the Centers for Disease Control: 12 ounces of beer, 8 ounces of malt liquor, 5 ounces of wine, or 1.5 ounces of liquor [30]. Based on the standard measures of alcohol, the serving size in grams for each of the categories was measured using commonly available samples of each type of beverage. Because estimates of nitrates and nitrites in alcoholic beverages were rarely reported in the literature, these compounds were not estimated for standard servings of these beverages. However, nitrosamine values were available and were used to calculate the estimated amount per serving using the same procedures used in the food calculations.

## Results

Table 2 displays the calculated amount per serving for food items grouped into dairy products; fruit; grains; meat and beans; vegetables; fats, oils, nuts, and sweets; and alcoholic beverages. The estimates in Table 2 are based on standard serving sizes for adults. Nitrate values ranged from 0 – 188.999 mg/serving with the highest concentrations occurring in vegetable products. Spinach and squash contain the highest amounts of nitrate per serving with values of 188.999 and 43.608 mg respectively. Sweets, nuts, fats and oils contain very little nitrates per serving.

**Table 2:** Estimates of nitrates, nitrites and nitrosamines in food items and alcoholic beverages by the National Birth Defects Prevention Study calculated serving size

**Food Item**	Serving Size g	Nitrates mg/serving	Nitrites mg/serving	Nitrosamines μg/serving
***Dairy Products***				
Cheese (1 slice or 1 oz)	28	0.400	0.023	0.066
Cottage or ricotta cheese (1/2 cup)	113	0.260	0.000	0.266
Ice cream (1/2 cup)	72	0.209	0.038	0.031
Skim or low fat milk (8 oz glass)	244	0.862	0.011	0.209
Whole milk (8 oz glass)	244	0.212	0.001	0.065
Yogurt (1 cup)	245	0.333	0.071	0.002
***Fruit Products***				
Avocado (1) or guacamole (1 cup)	201	5.278	0.042	0.010
Bananas (1)	114	2.280	0.043	0.006
Cantaloupe (1/4 melon)	134	12.730	0.051	0.006
Fresh apples or pears (1)	138	1.436	0.014	0.007
Hawaiian punch, lemonade, or other fruit drinks (1 glass)	244	2.367	0.000	0.000
Orange juice (small glass)	186	3.720	0.000	0.000
Oranges (1)	184	3.680	0.000	0.000
Other fruits fresh, frozen, or canned (1/2 cup)	142	3.728	0.030	0.007
Peaches, apricots, plums, or nectarines (1 or 1/2 cup)	87	0.564	0.014	0.004
Raw chile peppers, jalapeño (1)	14	0.742	0.009	0.000
Salsa (1 cup) (fruit or tomato)	259	9.094	0.140	0.000
Tomatoes (1) or tomato juice (small glass)	123	3.953	0.054	0.000
***Grain Products***				
Biscuit, scone, croissant and muffin (1)	80	0.328	0.032	0.012
Cereal	30	0.138	0.039	0.012
Dark bread (slice) including wheat pita bread	25	0.213	0.030	0.000
Rice or pasta (1 cup)	140	2.305	0.303	0.000
Tortilla (1)	19	0.304	0.031	0.002
White bread (slice), pita bread, bagels and crackers	25	0.410	0.042	0.002
***Meat and Bean Products***				
Bacon (2 slices)	16	1.449	0.467	0.219
Beans or lentils, baked or dried (1/2 cup)	131	1.179	0.341	0.000
Beans, refried (1 cup)	252	2.268	0.655	0.000
Beef, pork, lamb or cabrito as a main dish (4–6 oz)	140	8.188	1.840	0.453
Beef, pork, lamb or cabrito sandwich or mixed dish	100	5.849	1.314	0.324
Chicken livers (1 oz)	28	0.958	0.571	0.007
Chicken or turkey (4–6 oz)	88	0.583	0.478	0.086
Eggs (1)	58	0.311	0.095	0.000
Fish (3–6 oz)	112	1.027	0.349	0.222
Hamburger (1 patty)	77	6.102	0.193	0.071
Hot dogs (1)	45	11.565	1.220	0.128
Liver, non specific (3–4 oz)	82	10.325	1.608	0.022
Organ meats and tongue (3–4 oz)	85	0.882	0.480	0.062
Peas or lima beans (1/2 cup frozen, canned)	80	2.634	0.070	0.000
Processed meats – sausage, salami, lunchmeat, pâté (piece or slice)	28	1.700	0.314	0.124
***Vegetable Products***				
Broccoli (1/2 cup)	78	26.607	0.251	0.000
Cabbage, cauliflower, brussel sprouts (1/2 cup)	75	12.976	0.143	0.000
Carrots, cooked (1/2 cup)	78	13.013	0.115	0.000
Carrots, raw (1/2 cup or 2–4 sticks)	36	6.006	0.053	0.000
Corn (1 ear or 1/2 cup frozen, canned)	82	3.690	0.164	0.000
Potatoes baked, boiled (1) or mashed (1 cup)	156	22.673	0.164	0.000
Soy milk or soy yogurt (8 oz)	244	13.333	0.220	0.000
Spinach or collard greens, cooked (1/2 cup)	90	188.999	0.177	0.000
Squash (1/2 cup)	103	43.608	0.082	0.000
String beans (1/2 cup)	68	11.767	0.128	0.000
Tofu, tempeh or soy burgers (4oz)	113	6.174	0.102	0.000
Yams or sweet potatoes (1/2 cup)	68	3.128	0.048	0.000
***Fats, Oils, Nuts, and Sweets***				
Butter (pat), added to food or bread	5	0.000	0.000	0.000
Cake (slice) or donut (1)	64	1.600	0.083	0.000
Candy without chocolate (1 oz)	28	0.000	0.000	0.010
Chocolate (1 oz)	21	0.000	0.000	0.010
Cookies (1)	25	0.092	0.011	0.002
French fried potatoes (4 oz)	112	4.144	0.078	0.000
Margarine (pat), added to food or bread	5	0.002	0.000	0.000
Nuts (small packet or 1 oz)	18	0.104	0.006	0.000
Oil and vinegar dressing (1 tbs)	15	0.000	0.000	0.000
Peanut butter (1 tbs)	16	0.093	0.006	0.000
Pie (slice)	125	2.338	0.138	0.000
Potato or corn chips (small bag or 1oz)	28	1.049	0.020	0.000
***Alcoholic Beverages***				
Beer (12 fl. oz/354.9 ml)	357	-	-	0.531
Wine (5 fl. oz/147.9 ml)	136	-	-	0.019
Liquor and mixed drinks (1.5 fl. oz/44.4 ml)	41	-	-	0.027
Malt beverages (8 fl. oz/236.6 ml)	245	-	-	0.301

Nitrite values range from 0 – 1.840 mg/serving with the highest concentrations occurring in meat and bean products. Beef, pork, lamb, or *cabrito *(goat meat) as a main dish and liver contain the highest amounts of dietary nitrite per serving with 1.840 and 1.608 mg respectively. Negligible sources of dietary nitrite are found in cottage cheese, fats such as butter or margarine, and various fruit juices.

Nitrosamine values from food items ranged from 0 – 0.453 μg/serving with the highest concentrations occurring in meat and dairy products. Beef, pork, lamb, or *cabrito *as a sandwich or main dish contains 0.324 and 0.453 μg/serving. Cottage or ricotta cheese, fish and bacon contain high levels of nitrosamines with 0.266, 0.222 and 0.219 μg/serving respectively. Alcoholic beverages also contain high levels of nitrosamines, with beer and malt beverages containing the highest amount of nitrosamines per serving at 0.531 μg and 0.301 μg respectively. Wine and liquor contain relatively little nitrosamines per serving with values of 0.019 and 0.027 μg respectively. Fruits, vegetables, sweets, and fats do not contain significant amounts of nitrosamines per serving.

Additional file 1, Food database information, lists the reported nitrate, nitrite, and nitrosamine values of the food items from the literature, and those items for which substitutions or calculations were necessary based on the available data. These values were used to calculate the summary estimates of nitrate, nitrite, and nitrosamine content per food item. The food items are grouped into categories consisting of dairy products; fruit; grains; meat and beans; vegetables; fats, oils, nuts, and sweets; and alcoholic beverages.

## Discussion

Based on our estimates, total dietary nitrate intake per serving is most heavily influenced by vegetable consumption, specifically the green leafy varieties. However, nitrate intake in conjunction with vitamin C and possibly vitamin E may inhibit endogenous nitrosamine formation [31]. Fruits and vegetables are sources of vitamins, minerals, and dietary fiber, including pectin. Wawrzyniak found that pectin rich diets increase the total number of *Enterobacteriaceae *in the stomach of rats, which is associated with the reduction of nitrates to nitrites, but also noted that pectin was responsible for decreasing the amount of sodium nitrite present under normal gastric conditions *in vitro *[32,33]. The benefits of fruit and vegetable consumption with their vitamin content most likely negate the potential harmful effects of nitrate intake from these sources. Our estimates indicate that nitrites and nitrosamines are most commonly associated with the consumption of meats, processed meats and fish. Based on our results, beer had the largest estimated amount of nitrosamine per serving.

Potential limitations in our study include sources of error associated with food item substitutions; different methodologies for measuring nitrates, nitrites, and nitrosamines in food; estimate calculations; and changes in concentration of these compounds in foods over time. The variability of nitrates, nitrites, and nitrosamines in food items may also be a source of error. The scarcity of reported nitrate and nitrite values for alcoholic beverages makes it difficult to create relevant estimates. Potentially, the nitrate/nitrite content of water used to produce non-distilled beverages could be used to estimate these compounds; however, this information was not available. Additionally, potential error stems from our substitutions and recipes used when the exact value for a food item was not available in the literature, although we expect the impact of these substitutions to be negligible. It is possible that nitrate, nitrite, and nitrosamine content in food items may vary by period measured, preparation, and analytical techniques used. To generate the most accurate estimate possible, we weighted individual values by their sample size. In most cases, weighting the values by the sample size more accurately reflects the available data. However, in a few cases, the estimate generated may be less accurate because of the over representation of values with extremely large samples sizes. Our study was also limited based on the relatively small number of references that were relevant to the current U.S. population based on year of publication and country of origin. Of the 30 references used to calculate estimates, only 11 were published since 1990 and none of those were based on U.S. or Canadian values. Therefore, estimates of nitrates, nitrites, and nitrosamines should be updated with the appropriate analyses of food products currently available in the United States. Consideration should also be given to regional and ethnic differences in food availability and dietary intake.

Despite these limitations, our study also has several strengths. The estimates we generated are unique in that they were created to be used with the WFFQ, a commonly used research tool to assess dietary intake in research studies. Intake of nitrates, nitrites, and nitrosamines has likely changed over time because of procedural changes in food preservation and dietary behavioral changes [34]. Articles in the literature, such as those authored by Jakszyn *et al*. and Cornee *et al*., provide values of nitrate, nitrite and nitrosamines in foods from the global literature; however, we were unable to locate summary estimates for the U.S. population that systematically accounted for time and country of origin [10,35]. In the present study, we compiled, ranked, selected, and weighted the reported values to generate an estimate for each food item. Our estimates are not intended to be comprehensive in terms of all published literature regarding nitrate, nitrite, and nitrosamines in foods. Rather, the estimates were generated to reflect relevant information in the published literature that was representative of the U.S. population with respect to time and country of origin.

## Conclusion

Laboratory analysis of nitrate, nitrite, and nitrosamine content in food items commonly available in the United States should be conducted to determine current exposures to the U.S. population. In the absence of current data based on laboratory analysis, our estimates can be used to assess dietary exposures to these compounds. To the best of our knowledge, our estimates are the only ones published to date that are developed specifically for use with the WFFQ and intended for use with the U.S. population. The estimates provided may also be useful in other research studies, specifically in exploring the relation between intake of *N*-nitroso compounds and their precursors with health conditions such as cancer and adverse pregnancy outcomes.

## Competing interests

The authors declare that they have no competing interests.

## Authors' contributions

JG carried out the literature review, compiled the food database, calculated dietary estimates and prepared the manuscript. MS assisted with the literature review, compiled food database, calculated dietary estimates and assisted with the manuscript preparation. JH assisted with the statistical analysis and provided contribution to the methods section of the manuscript. JS contributed to the nutritional aspects of the manuscript. AR contributed to the discussion section of the manuscript. JB conceived of the study, supervised the analysis, and contributed to the manuscript discussion. All authors read and approved the final manuscript.

## Supplementary Material

Additional file 1 **Supplemental file 1**. Food database information, lists the reported nitrate, nitrite, and nitrosamine values of the food items from the literature, and those items for which substitutions or calculations were necessary based on the available data. These values were used to calculate the summary estimates of nitrate, nitrite, and nitrosamine content per food item. The food items are grouped into categories consisting of dairy products; fruit; grains; meat and beans; vegetables; fats, oils, nuts, and sweets; and alcoholic beverages. Click here for file
