# Supplementary material for: Development of estimates of dietary nitrates, nitrites, and nitrosamines for use with the short willet food frequency questionnaire
Source: Nutr J. 2009 Apr 6;8:16. doi: 10.1186/1475-2891-8-16 (PMC2669451; doi:10.1186/1475-2891-8-16)
Supplement: Additional file 1 — Supplemental file 1. Food database information, lists the reported nitrate, nitrite, and nitrosamine values of the food items from the literature, and those items for which substitutions or calculations were necessary based on the available data. These values were used to calculate the summary estimates of nitrate, nitrite, and nitrosamine content per food item. The food items are grouped into categories consisting of dairy products; fruit; grains; meat and beans; vegetables; fats, oils, nuts, and sweets; and alcoholic beverages. [file 1475-2891-8-16-S1.pdf]

Supplemental file 1: Nitrate, nitrite, and nitrosamine content of food items listed in the food database and reference information used to generate the summary estimates by category

Reported nitrate content of food items listed in the food database and reference information used to generate the summary estimates by category.

NBDPS Food Item	Food Name	Mean[a] (mg/100g)	Value Type[b]	Range[c]	Sample Size[c]	Author	Rank
**Dairy Products**							
Cheese	Cheese	1.610	mn	0.12-5.6	130	French National Inventory	2
	Cheese (Camembert)	1.600	mn	0.24-4.05	30	French National Inventory	2
	Cheese (Holland)	2.400	mn	0.74-5.6	10	French National Inventory	2
	Cheese (Pyrenees)	0.400	mn	0.12-1.2	10	French National Inventory	2
	Cheese (Sant-Nectaire)	1.800	mn	0.12-5.50	30	French National Inventory	2
	Cheese (spread)	1.700	mn	0.43-4.3	30	French National Inventory	2
	Cheese	0.050	w			Howe et al.	1
	Cream cheese	0.050	mn			Pobel et al.	2
	Hard cheese	1.700	mn			Pobel et al.	2
	Soft cheese	1.600	mn			Pobel et al.	2
	Creamed cheese	0.025	mn	<0.50	4	Thomson and Swallow	2
	Cheese	0.150	mn	<0.3	31	Von Collet	2
Cottage or ricotta cheese	Cottage Cheese	0.230	mn	<0.5-0.9	4	Thomson and Swallow	2
Ice cream[d]	Milk, sugar, eggs	0.29	mn				
Skim or low fat milk	Milk	0.460	mp	0.39-0.53	20	Food Standards Agency	2
	Milk (skim milk)	0.050	mn			Pobel et al.	2
	Milk	0.150	mp	<0.3	9	Von Collet	2
Whole milk	Milk	0.460	mp	0.39-0.53	20	Food Standards Agency	2
	Milk (whole)	0.050	w	tr-0.34	217	Mahieu et al.	2
	Milk	0.150	mp	<0.3	9	Von Collet	2
Yogurt	Yogurt	0.050	mn			Pobel et al.	2
	Yogurt	0.150	mp	<0.3	6	Von Collet	2
**Fruit Products**							
Avocado or guacamole	Fruit	2.700	mn	1.2-4.6	20	Food Standards Agency	2
	Fruit	2.000	w			Natl. Academy of Sciences	3
	Fresh fruit	2.000	mn			Pobel et al.	2
Bananas	Bananas	2.000	w			Cornee	2
Cantaloupe	Watermelon	9.500	mn		1	Tamme	5
Fresh apples or pears	Pear	0.390	mn	<0.3-1.08	24	Von Collet	2
	Apple	1.750	mn	<0.3-10.8	22	Von Collet	2
Fruit drinks	Fruit juices	0.970	w			Howe et al.	1
Orange juice	Oranges	2.000	w			Howe et al.	1
Oranges	Oranges	2.000	w			Howe et al.	1
Other fruits	Fruit	2.700	mn	1.2-4.6	20	Food Standards Agency	2
	Fruit	2.000	w			Natl. Academy of Sciences	3
	Fresh fruit	2.000	mn			Pobel et al.	2
	Grapes	0.810	mn	<0.3-3.40	23	Von Collet	2

	Strawberry	7.170	mn	2.43-15.34	9	Von Collet	2
Peaches, apricots, plums, or nectarines	Peach	0.780	mn	<0.3-3.13	13	Von Collet	2
	Apricot	0.600	mn	<0.3-0.95	3	Von Collet	2
	Nectarine	0.150	mn	<0.3-0.89	5	Von Collet	2
	Plum	0.880	mn	<0.3-2.03	4	Von Collet	2
Raw chile peppers	Pepper (sweet)	12.000	w			Natl. Academy of Sciences	3
	Pepper (sweet green)	6.200	mn			Siciliano et al.	3
	Peppers (sweet)	5.000	mn		3	Siciliano et al.	3
Salsa (fruit or tomato)[d]	Tomato, peppers, onion	3.51	mn				
Onion[e]	Onion	4.800	mn		8	Food Standards Agency	2
	Onion	2.300	mn		63	French National Inventory	2
	Onion, chopped	3.300	mn		1	Siciliano et al.	3
	Onion, whole	12.800	mn		1	Siciliano et al.	3
	Onion	5.500	mn		21	Tamme	5
	Onion	8.000	mn			Thomson and Swallow	2
	Onion	4.800	mn			Thomson and Swallow	2
	Onion	2.300	mn			Thomson and Swallow	2
	Onion	5.110	mn		4	Von Collet	2
Tomatoes or tomato juice	Tomato	1.700	mn	0.4-4.2	4	Food Standards Agency	2
	Tomato	3.300	mn	0.14-58.9	62	French National Inventory	2
	Tomatoes	5.800	w			Howe et al.	1
	Tomato	5.800	w			Natl. Academy of Sciences	3
	Tomato	3.080	mn			Pobel et al.	2
	Tomato	3.020	mn	<0.3-8.94	9	Von Collet	2
**Grain Products**							
Biscuits, scones, croissants and muffins[d]	Four, butter, milk	0.41	mn				
Cereal	Cereal	2.500	w			Cornee	2
	Cereals	0.105	mp	0.10-0.11	20	Food Standards Agency	2
	Cereal, cooked	1.200	w			Howe et al.	1
	Cereal	0.940	mn	0.68-1.16	9	Von Collet	2
Dark bread	Wheat flour	0.850	mn			Pobel et al.	2
Rice or pasta	Rice, non specific	1.200	w			Howe et al.	1
	Pasta	1.240	w			Howe et al.	1
	Pasta, rice	2.500	mn			Pobel et al.	2
Tortilla	Bread (non specific)	0.760	mp	0.72-0.8	20	Food Standards Agency	2
	Bread	2.500	w	0.64-6.14	20	French National Inventory	2
	Bread (non specific)	1.200	w			Howe et al.	1
	White bread	2.500	mn			Pobel et al.	2
White bread	Bread (non specific)	0.760	mp	0.72-0.8	20	Food Standards Agency	2
	Bread	2.500	w	0.64-6.14	20	French National Inventory	2
	Bread (non specific)	1.200	w			Howe et al.	1
	White bread	2.500	mn			Pobel et al.	2
**Meat and Bean Products**							
Bacon	Bacon	10.110	mn	0.14-44.47	57	Food Standards Agency	2

	Bacon	3.200	w			Howe et al.	1
	Bacon	3.650	mn	<0.5-8.1	10	Thomson and Swallow	2
Beans or lentils	Black-eyed peas	0.900	mn		2	Siciliano et al.	3
Beans, refried	Black-eyed peas	0.900	mn		2	Siciliano et al.	3
Beef, pork, lamb[g]							
Pork sub group	Ham	15.700	w	0.30-92.1	37	Cornee	2
	Pork (shoulder)	10.170	mn	0.83-27.99	3	Food Standards Agency	2
	Ham	7.170	mn	0.03-44.59	57	Food Standards Agency	2
	Ham (chopped)	3.910	mn	0.33-14.22	8	Food Standards Agency	2
	Ham (mountain)	16.300	mn	0.37-81.15	17	French National Inventory	2
	Pork	4.950	w			Howe et al.	1
	Ham	15.660	mn			Pobel et al.	2
	Pork	0.740	mn			Saccani and Tanzi	2
	Pork shoulder	0.510	mn	0.36-1.02	15	Saccani and Tanzi	2
	Pork loin	0.390	mn	0.36-1.02	11	Saccani and Tanzi	2
	Dry cured ham	4.830	mn	0.17-10.4	13	Saccani and Tanzi	2
	Ham	1.660	mn	<0.5-3.2	10	Thomson and Swallow	2
Beef sub group	Beef (corned)	2.160	mn	0.44-7.0	11	Food Standards Agency	2
	Beef	5.180	mn	2.1-8.25	2	Food Standards Agency	2
	Corned beef	14.160	w			Howe et al.	1
	Beef	0.380	mn			Saccani and Tanzi	2
	Beef filet	0.460	mn	0.24-0.92	12	Saccani and Tanzi	2
	Beef mince	0.370	mn	<0.5-1.5	4	Thomson and Swallow	2
	Corned silverside	1.810	mn	0.7-3.6	10	Thomson and Swallow	2
Meat sub group	Meat	1.000	w			Natl. Academy of Sciences	3
	Red meat	1.000	mn			Pobel et al.	2
	Smoked Meat	13.930	mn			Pobel et al.	2
Chicken livers	Pâté (chicken liver)	3.420	mn	0.45-5.36	4	Food Standards Agency	2
Chicken or Turkey							
Chicken sub group	Chicken	0.820	mn	0.59-1.25	3	Food Standards Agency	2
	Chicken	0.510	mn	0.26-0.89	10	Saccani and Tanzi	2
Turkey sub group	Turkey	0.750	mn	0.05-1.39	6	Food Standards Agency	2
Poultry sub group	Poultry	0.830	mp	0.81-0.85	20	Food Standards Agency	2
	Poultry	1.000	mn			Pobel et al.	2
Eggs	Egg	0.490	mp	0.44-0.54	20	Food Standards Agency	2
	Egg	1.000	w			Howe et al.	1
	Egg	1.000	mn			Pobel et al.	2
Fish	Fish, smoked	1.000	mn	0.88-5.76	5	Cornee	2
	Fish	1.200	w	0.1-4.1		Cornee	2
	Fish	0.100	w	0.88-5.76	5	Cornee	2
	Fish	1.100	mn	0.5-1.9	20	Food Standards Agency	2
	Fish, other	1.000	w			Howe et al.	1
	Fish, smoked	0.190	w			Howe et al.	1
	Fresh fish	1.180	mn			Pobel et al.	2
	Smoked Fish	1.030	mn			Pobel et al.	2

Hamburger	Hamburger	7.900	mn	0.6-21.1	4	Thomson and Swallow	2
Hot dogs	Frankfurt	25.700	mn	6.14-186.2	10	French National Inventory	2
Liver, non-specific	Sausage (liver)	9.370	mn	2.68-19.02	5	Food Standards Agency	2
	Pâté (liver)	18.300	mn	0.55-42.3	5	French National Inventory	2
	Liver, beef	1.000	w			Howe et al.	1
Organ meats and tongue							
Tongue sub group	Tongue	1.590	mn	<0.002-5.5	20	Food Standards Agency	2
Offals sub group	Offals	0.460	mp	0.39-0.53	20	Food Standards Agency	2
	Offals	1.000	mn			Pobel et al.	2
Peas or lima beans	Peas	1.100	mn	0.12-3.1	18	French National Inventory	2
	Peas	2.800	w			Natl. Academy of Sciences	3
	Lima beans	5.400	w			Natl. Academy of Sciences	3
	Peas	1.010	mn			Pobel et al.	2
	Pea pods (Chinese)	1.300	mn		2	Siciliano et al.	3
	Green peas	2.000	mn		4	Siciliano et al.	3
	Peas	0.600	mn		3	Siciliano et al.	3
Processed meats							
Sausage sub group	Sausage (liver)	9.370	mn	2.68-19.02	5	Food Standards Agency	2
	Sausage	17.600	mn	0.72-115.8	10	French National Inventory	2
	Sausage (garlic)	24.800	mn	1.78-56.2	10	French National Inventory	2
	Sausage	18.100	mn	0.18-154.5	20	French National Inventory	2
	Saucisson	3.300	mn	0.15-18.4	24	French National Inventory	2
	Sausage (beef & pork)	9.790	w			Howe et al.	1
	Cured meat (sausage)	13.600	mn			Pobel et al.	2
	Dry cured sausage	1.550	mn	0.14-7.69	24	Saccani and Tanzi	2
	Saveloys (red sausage)	2.850	mn	0.8-6.3	10	Thomson and Swallow	2
	Beef sausages	0.180	mn	<0.5-1.8	10	Thomson and Swallow	2
Salami sub group	Salami	2.480	mn	<0.5-5.6	10	Thomson and Swallow	2
Lunchmeat sub group	Luncheon meat	2.070	mn	0.64-4.88	19	Food Standards Agency	2
	Luncheon	3.090	mn	2.2-5.3	10	Thomson and Swallow	2
Pâté sub group	Pâté (chicken liver)	3.420	mn	0.45-5.36	4	Food Standards Agency	2
	Pâté (liver)	18.300	mn	55-42.3	5	French National Inventory	2
	Pâté	2.100	mn	1.35-2.64	5	French National Inventory	2
Preserved meat sub group	Preserved meat	8.100	mn			Pobel et al.	2
**Vegetable Products**							
Broccoli	Broccoli	74.000	w			Natl. Academy of Sciences	3
	Broccoli (spears)	46.400	mn		6	Siciliano et al.	3
	Broccoli (chopped)	57.300	mn		4	Siciliano et al.	3
	Broccoli	13.300	mn	5.1-28	8	Thomson and Swallow	2
Cabbage, cauliflower, or Brussels sprouts							
Cabbage sub group	Cabbage	33.800	mn		16	Food Standards Agency	2
	Cabbage	16.000	mn	0.25-79.2	94	French National Inventory	2
	Kale	33.000	mn	7.7-58.4	30	French National Inventory	2
	Cabbage	52.000	w			Howe et al.	1
	Kale	80.000	w			Natl. Academy of Sciences	3

	White cabbage	39.500	mn		40	Petersen and Stoltze	2
	Chinese cabbage	100.000	mn		30	Petersen and Stoltze	2
	Cabbage	15.210	mn			Pobel et al.	2
	Cabbage	78.400	mn			Siciliano et al.	3
	Kale	277.000	mn		3	Siciliano et al.	3
	Kale	160.000	mn		3	Siciliano et al.	3
	Sauerkraut	6.800	mn		1	Siciliano et al.	3
	Silverbeet	74.000	mn	19.0-169.0	8	Thomson and Swallow	2
	Cabbage	33.100	mn	12.0-69.0	8	Thomson and Swallow	2
	Cabbage	26.390	mn	2.90-63.62	16	Von Collet	2
Cauliflower sub group	Cauliflower	8.600	mn		8	Food Standards Agency	2
	Cauliflower	10.700	mn	0.3-77.1	53	French National Inventory	2
	Cauliflower	48.000	w			Natl. Academy of Sciences	3
	Cauliflower	25.400	mn		5	Siciliano et al.	3
Brussels sprouts sub group	Brussels sprouts	5.900	mn		4	Food Standards Agency	2
	Brussels sprouts	2.200	mn	0.15-9.7	30	French National Inventory	2
	Brussels sprouts	12.000	w			Natl. Academy of Sciences	3
	Brussels sprouts	8.400	mn		7	Siciliano et al.	3
Carrots (cooked, raw)[h]	Carrot	9.700	w	1.5-11.5	20	Food Standards Agency	2
	Carrot	18.400	mn	0.12-137.2	80	French National Inventory	2
	Carrots	20.000	w			Howe et al.	1
	Carrot	20.000	w			Natl. Academy of Sciences	3
	Carrot	17.620	mn			Pobel et al.	2
	Carrot	7.200	mn		8	Siciliano et al.	3
	Carrot	9.700	mn		5	Siciliano et al.	3
	Carrot	20.500	mn		18	Siciliano et al.	3
	Carrot	5.830	mn	<0.5-29.0	8	Thomson and Swallow	2
	Carrot	28.810	mn	3.51-84.16	7	Von Collet	2
Corn	Corn	4.500	w			Natl. Academy of Sciences	3
	Corn	4.500	mn		3	Siciliano et al.	3
Potatoes	Potatoes	15.500	mn	0.3-107.7	180	Food Standards Agency	2
	Potatoes (white)	14.400	mn	0.12-119.1	116	French National Inventory	2
	Potatoes, non specific	11.000	w			Howe et al.	1
	Potato	11.000	mn	0.7-30.4	40	Petersen and Stoltze	2
	Potatoes	14.220	mn			Pobel et al.	2
	Potatoes (whole, small)	15.000	mn		1	Siciliano et al.	3
	Potatoes (whole)	6.300	mn		2	Siciliano et al.	3
	Potatoes (sliced)	6.900	mn		1	Siciliano et al.	3
	Potatoes	12.900	mn	4.8-24.0	8	Thomson and Swallow	2
Soy milk or soy yogurt	Soybean sprouts	5.600	mn		40	Chung et al.	5
	Miso and Shoyu	0.030	mn			Maki et al. 35	5
Spinach or collard greens							
Spinach sub group	Lettuce	105.100	mn		22	Food Standards Agency	2
	Spinach	163.100	mn		13	Food Standards Agency	2
	Spinach	44.300	mn	3.6-114.9	41	French National Inventory	2
	Lettuce	268.400	mp	5.8-540.6	159	Merino et al.	2

	Lettuce	93.100	mp	9.4-229.8	71	Merino et al.	2
	Spinach	174.700	mp	4.7-597.5	63	Merino et al.	2
	Spinach	55.100	mp	21.3-186.2	70	Merino et al.	2
	Lettuce	82.600	mp	44.2-203.8	14	Merino et al.	2
	Lettuce	170.000	w			Natl. Academy of Sciences	3
	Spinach	180.000	w			Natl. Academy of Sciences	3
	Lettuce	170.000	w			Natl. Academy of Sciences	3
	Lettuce	107.400	mn			Petersen and Stoltze	2
	Spinach	68.000	mn	9.0-151.0	20	Petersen and Stoltze	2
	Spinach	178.300	mn	4.8-563.0	36	Petersen and Stoltze	2
	Lettuce	244.000	mn		122	Petersen and Stoltze	2
	Lettuce	136.280	mn			Pobel et al.	2
	Spinach	44.260	mn			Pobel et al.	2
	Spinach	222.000	mn		7	Siciliano et al.	3
	Lettuce	110.000	mn			Siciliano et al.	3
	Spinach	214.000	mn		4	Siciliano et al.	3
	Spinach	57.300	mn		1	Siciliano et al.	3
	Lettuce	159.000	mn	8.3-342.0	18	Thomson and Swallow	2
	Spinach	99.000	mn	10.0-156.0	8	Thomson and Swallow	2
	Spinach	154.970	mn	53.3-203.9	6	Von Collet	2
	Lettuce	186.810	mn	6.5-501.95	28	Von Collet	2
Collard Greens sub group	Collard greens	245.000	mn		4	Siciliano et al.	3
	Mustard greens	239.000	mn		4	Siciliano et al.	3
	Turnip greens	346.000	mn		3	Siciliano et al.	3
	Collard greens	264.000	mn		2	Siciliano et al.	3
	Turnip greens	223.000	mn		2	Siciliano et al.	3
	Mustard greens	136.000	mn		2	Siciliano et al.	3
Squash	Squash (acorn)	3.400	mn			Siciliano et al.	3
	Squash (butternut)	67.800	mn			Siciliano et al.	3
	Squash (zucchini)	66.500	mn			Siciliano et al.	3
	Squash	16.000	mn		2	Siciliano et al.	3
	Zucchini	53.300	mn		4	Siciliano et al.	3
String Beans	Green beans	15.040	mn			Pobel et al.	2
	Green beans	27.000	mn		4	Siciliano et al.	3
	Green beans	10.000	mn		5	Siciliano et al.	3
Tofu, tempeh or soy burgers	Soybean sprouts	5.600	mn		40	Chung et al.	5
	Miso and Shoyu	0.030	mn			Maki et al.	5
Yams or sweet potatoes	Sweet potatoes	4.600	w			Natl. Academy of Sciences	3
**Fats, Oils, Nuts, and Sweets**							
Butter	Butter and cream	0.000	mn			Pobel et al.	2
Cake or donut	Cake	2.500	mn			Pobel et al.	2
Candy without chocolate	Sugar, sweets	0.000	mn			Pobel et al.	2
Chocolate	Sugar, chocolate, sweets	0.000	mn			Pobel et al.	2
Cookies[d]	Sugar, butter, milk, eggs, flour	0.370	mn				
French fried potatoes	Potatoes (hash brown)	3.700	mn		2	Siciliano et al.	3

Margarine	Margarine	0.050	w			Howe et al.	1
Nuts	Nuts	0.580	mp	0.55-0.61	20	Food Standards Agency	2
Oil and Vinegar dressing	Salad Dressing	0.000	mn			Howe et al.	2
	Oil	0.000	mn			Pobel et al.	2
Peanut butter	Nuts	0.580	mp	0.55-0.61	20	Food Standards Agency	2
Pie	Pie	1.870	mn			Howe et al.	1
Potato chips or corn chips	Potatoes (hash brown)	3.700	mn		2	Siciliano et al.	3

[a] Mean is either the average, weighted mean, midpoint, or half the detection limit when reported as less than the detection limit as listed under nitrate value type.

[b] mn=mean, mp=mid point, w = weighted mean

[c] Information provided when reported in the literature; tr = trace amount

[d] recipe calculation

[e] item used in recipe calculation for salsa

[f] Beef, pork, lamb as a sandwich or mixed dish and beef, pork, lamb as a main dish are made up of the same values, only the serving size is different.

[g] Carrots raw or cooked are made up of the same values, only the serving size is different.

Reported nitrite content of food items listed in the food database and reference information used to generate the summary estimates by category.

NBDPS Food Item	Food Name	Mean[a] (mg/100g)	Value Type[b]	Range[c]	Sample Size[c]	Author	Rank
**Dairy Products**							
Cheese	Cheese	0.079	mn	0.01-0.36	130	French National Inventory	2
	Cheese (Camembert)	0.170	mn	0.07-0.60	30	French National Inventory	2
	Cheese (Holland)	0.060	mn	0.03-0.13	10	French National Inventory	2
	Cheese (Pyrenees)	0.040	mn	0.02-0.07	10	French National Inventory	2
	Cheese (Sant-Nectaire)	0.107	mn	0.01-0.29	30	French National Inventory	2
	Cheese (spread)	0.100	mn	0.03-0.27	30	French National Inventory	2
	Cheese	0.000	w			Howe et al.	1
	Cream cheese	0.000	mn			Pobel et al.	2
	Hard cheese	0.100	mn			Pobel et al.	2
	Soft cheese	0.070	mn			Pobel et al.	2
	Creamed cheese	0.025	mn	<1.0	4	Thomson and Swallow	2
	Cheese	0.010	mn	<0.02	31	Von Collet	2
Cottage or ricotta cheese	Cottage cheese	0.000	mn	<1.5	4	Thomson and Swallow	2
Ice cream[d]	Milk, sugar, eggs	0.053	mn				
Skim or low fat milk	Milk	0.002	mp	0-0.004	20	Food Standards Agency	2
	Milk	0.000	mn			Pobel et al.	2
	Milk	0.010	mn	<0.02	9	Von Collet	2
Whole milk	Milk	0.002	mp	0-0.004	20	Food Standards Agency	2
	Milk	0.000	w	tr-0.01	450	Mahieu et al.	2
	Milk	0.010	mn	<0.02	9	Von Collet	2
Yogurt	Yogurt	0.000	mn			Pobel et al.	2
	Yogurt	0.034	mp	0.01-0.05	6	Von Collet	2
**Fruit Products**							
Avocado or guacamole	Fruit	0.040	mn	<0.04-0.05	20	Food Standards Agency	2
	Fruit	0.000	w			Natl. Academy of Sciences	3
	Fresh fruit	0.000	mn			Pobel et al.	2
Bananas	Fruit	0.040	mn	<0.04-0.05	20	Food Standards Agency	2
	Fruit	0.000	w			Natl. Academy of Sciences	3
	Fresh fruit	0.000	mn			Pobel et al.	2
Cantaloupe	Fruit	0.040	mn	<0.04-0.05	20	Food Standards Agency	2
	Fruit	0.000	w			Natl. Academy of Sciences	3
	Fresh fruit	0.000	mn			Pobel et al.	2
Fresh apples or pears	Pear	0.010	mp	<0.02	24	Von Collet	2
	Apple	0.010	mp	<0.02	22	Von Collet	2
Fruit drinks	Fruit juices	0.000	w			Howe et al.	1
Orange juice	Oranges	0.000	w			Howe et al.	1
Oranges	Oranges	0.000	w			Howe et al.	1
Other fruits	Fruit	0.040	mn	<0.04-0.05	20	Food Standards Agency	2
	Fruit	0.000	w			Natl. Academy of Sciences	3

	Fresh fruit	0.000	mn			Pobel et al.	2
	Grapes	0.010	mp	<0.02	23	Von Collet	2
	Strawberry	0.010	mp	<0.02	9	Von Collet	2
Peaches, apricots, plums, or nectarines	Peach	0.010	mp	<0.02	13	Von Collet	2
	Apricot	0.010	mp	<0.02	13	Von Collet	2
	Nectarine	0.010	mp	<0.02	5	Von Collet	2
	Plum	0.065	mn	<0.02	4	Von Collet	2
Raw chile peppers	Pepper	0.040	w			Natl. Academy of Sciences	3
	Pepper	0.040	mn			Siciliano et al.	3
	Peppers	0.070	mn		3	Siciliano et al.	3
Salsa (fruit or tomato)[d]	Tomato, peppers, onions	0.054	mn			Food Standards Agency	2
Onion[e]	Onion	0.070	mn		63	French National Inventory	2
	Onion, chopped	0.100	mn		1	Siciliano et al.	3
	Onion, whole	0.040	mn		1	Siciliano et al.	3
	Onion	0.020	mn		4	Von Collet	2
Tomatoes or tomato juice	Tomato	0.050	mn	0.01-0.21	62	French National Inventory	2
	Tomatoes	0.000	w			Howe et al.	1
	Tomato	0.030	mn			Pobel et al.	2
	Tomato	0.010	mp	<0.02	9	Von Collet	2
**Grain Products**							
Biscuits, scones, croissants and muffins[d]	Flour, butter, milk	0.040	mn				
Cereal	Cereal	0.130	w			Cornee	2
	Cereals	0.180	mn	<0.04-0.08	20	Food Standards Agency	2
	Cereal, cooked	0.260	w			Howe et al.	1
	Cereal	0.010	mp	<0.02	9	Von Collet	2
Dark bread	Wheat flour	0.120	mn			Pobel et al.	2
Rice or pasta	Rice, non specific	0.260	w			Howe et al.	1
	Pasta	0.260	w			Howe et al.	1
	Pasta, rice	0.130	mn			Pobel et al.	2
Tortilla	Bread	0.200	mp	<0.02-0.4	20	Food Standards Agency	2
	Bread	0.130	w	0.01-0.67	20	French National Inventory	2
	Bread	0.260	w			Howe et al.	1
	White bread	0.130	mn			Pobel et al.	2
White bread	Bread	0.200	mp	<0.02-0.4	20	Food Standards Agency	2
	Bread	0.130	w	0.01-0.67	20	French National Inventory	2
	Bread	0.260	w			Howe et al.	1
	White bread	0.130	mn			Pobel et al.	2
**Meat and Bean Products**							
Bacon	Bacon	3.190	mn	<0.02-12.3	57	Food Standards Agency	2
	Bacon	0.700	w			Howe et al.	1
	Bacon	1.590	mn	<0.5-6.3	10	Thomson and Swallow	2
Beans or lentils	Black-eyed peas	0.260	mn		2	Siciliano et al.	3
Refried Beans	Black-eyed peas	0.260	mn		2	Siciliano et al.	3

Beef, pork, lamb[g]							
Pork sub group	Ham	1.580	w	0.07-184.0	37	Cornee	2
	Pork (shoulder)	5.350	mn	1.96-10.37	3	Food Standards Agency	2
	Ham	2.910	mn	0.22-16.97	57	Food Standards Agency	2
	Ham (chopped)	0.920	mn	0.06-2.69	8	Food Standards Agency	2
	Ham (mountain)	3.400	mn	0.07-18.40	17	French National Inventory	2
	Pork	0.720	w			Howe et al.	1
	Ham	1.580	mn			Pobel et al.	2
	Pork	0.070	mn			Saccani and Tanzi	2
	Dry cured ham	1.130	mn	0.18-1.94	13	Saccani and Tanzi	2
	Ham	1.990	mn	<0.5-11.9	10	Thomson and Swallow	2
Beef sub group	Beef (corned)	1.260	mn	<0.02-7.15	11	Food Standards Agency	2
	Beef	1.240	mn	0.42-2.06	2	Food Standards Agency	2
	Corned beef	1.940	w			Howe et al.	1
	Beef	0.080	mn			Saccani and Tanzi	2
	Beef mince	0.250	mp	<0.5	4	Thomson and Swallow	2
	Corned silverside	0.900	mn	<0.5-1.5	10	Thomson and Swallow	2
Meat sub group	Meat	0.100	w			Natl. Academy of Sciences	3
	Red meat	0.100	mn			Pobel et al.	2
	Smoked meat	1.200	mn			Pobel et al.	2
Chicken Livers	Pâté (chicken liver)	2.040	mn	0.5-5.55	4	Food Standards Agency	2
Chicken or Turkey							
Chicken sub group	Chicken	0.730	mn	0.11-1.90	3	Food Standards Agency	2
	Chicken	0.060	mn			Saccani and Tanzi	2
Turkey sub group	Turkey	0.530	mn	<0.02-2.0	6	Food Standards Agency	2
Poultry sub group	Poultry	0.045	mp	0.04-0.05	20	Food Standards Agency	2
	Poultry	0.100	mn			Pobel et al.	2
Eggs	Egg	0.170	mn		20	Food Standards Agency	2
	Egg	0.100	w			Howe et al.	1
	Egg	0.100	mn			Pobel et al.	2
Fish	Fish, smoked	0.900	mn	0.1-1.6	5	Cornee	2
	Fish	0.190	w	0-1.69		Cornee	2
	Fish	0.900	w	0.1-1.6	5	Cornee	2
	Fish	0.030	mp	nd-0.06	20	Food Standards Agency	2
	Fish, other	0.100	w			Howe et al.	1
	Fish, smoked	0.020	w			Howe et al.	1
	Fresh fish	0.100	mn			Pobel et al.	2
	Fish, smoked	0.900	mn			Pobel et al.	2
Hamburger	Hamburger	0.250	mp	<0.5	4	Thomson and Swallow	2
Hot dogs	Frankfurt	2.710	mn	0.27-13.6	10	French National Inventory	2
Liver	Sausage (liver)	3.430	mn	0.79-9.01	5	Food Standards Agency	2
	Pâté (liver)	0.890	mn	0.35-1.75	5	French National Inventory	2
	Liver, beef	0.100	w			Howe et al.	1
Organ meats and tongue							
Tongue sub group	Tongue	1.040	mn	0.2-3.13	20	Food Standards Agency	2
Offals sub group	Offals	0.090	mn	0.02-0.17	20	Food Standards Agency	2

	Offals	0.100	mn			Pobel et al.	2
Peas or lima beans	Peas	0.070	mn	0.007-.28	18	French National Inventory	2
	Peas	0.060	w			Natl. Academy of Sciences	3
	Lima beans	0.110	w			Natl. Academy of Sciences	3
	Peas	0.070	mn			Pobel et al.	2
	Pea pods	0.060	mn		2	Siciliano et al.	3
	Green peas	0.070	mn		4	Siciliano et al.	3
	Peas	0.040	mn		3	Siciliano et al.	3
Processed meats							
Sausage sub group	Sausage (liver)	3.430	mn	0.79-9.01	5	Food Standards Agency	2
	Sausage	0.940	mn	0.61-1.15	10	French National Inventory	2
	Sausage (garlic)	1.800	mn	0.23-3.53	10	French National Inventory	2
	Sausage	2.600	mn	0.07-15.2	20	French National Inventory	2
	Saucisson	0.920	mn	0.05-3.33	24	French National Inventory	2
	Sausage (beef and pork)	1.070	w			Howe et al.	1
	Cured meat (sausage)	1.260	mn			Pobel et al.	2
	Dry cured sausage	0.450	mn	0.21-1.60	24	Saccani and Tanzi	2
	Saveloys (red sausage)	3.560	mn	0.9-5.3	10	Thomson and Swallow	2
	Beef sausages	0.250	mp	<0.5		Thomson and Swallow	2
Salami sub group	Salami	0.740	mn	<0.5-3.6	10	Thomson and Swallow	2
Lunchmeat sub group	Luncheon meat	1.430	mn	0.13-5.56	19	Food Standards Agency	2
	Luncheon	2.460	mn	<0.5-7.1	10	Thomson and Swallow	2
Pâté sub group	Pâté (chicken liver)	2.040	mn	0.5-5.55	4	Food Standards Agency	2
	Pâté (liver)	0.890	mn	0.35-1.75	5	French National Inventory	2
	Pâté	0.400	mn	0.33-0.53	5	French National Inventory	2
Preserved meat sub group	Preserved meat	0.380	mn			Pobel et al.	2
**Vegetable Products**							
Broccoli	Broccoli	0.100	w			Natl. Academy of Sciences	3
	Broccoli (spears)	0.100	mn		6	Siciliano et al.	3
	Broccoli (chopped)	0.100	mn		4	Siciliano et al.	3
	Broccoli	0.600	mn	<0.5-2.7	8	Thomson and Swallow	2
Cabbage, cauliflower, or Brussels sprouts							
Cabbage sub group	Cabbage	0.110	mn	0.01-1.01	94	French National Inventory	2
	Kale	0.230	mn	0.05-0.93	30	French National Inventory	2
	Cabbage	0.050	w			Howe et al.	1
	Kale	0.100	w			Natl. Academy of Sciences	3
	White cabbage	0.016	mn		118	Petersen and Stoltze	2
	Chinese cabbage	0.034	mn		114	Petersen and Stoltze	2
	Cabbage	0.200	mn			Pobel et al.	2
	Cabbage	0.050	mn			Siciliano et al.	3
	Kale	0.180	mn		3	Siciliano et al.	3
	Kale	0.020	mn		3	Siciliano et al.	3
	Sauerkraut	0.040	mn		1	Siciliano et al.	3
	Silverbeet	0.250	mn	<0.5	8	Thomson and Swallow	2
	Cabbage	0.250	mn	<0.5	8	Thomson and Swallow	2

	Cabbage	0.120	mn	<.02-0.95	16	Von Collet	2
Cauliflower sub group	Cauliflower	0.440	mn	0.01-9.6	53	French National Inventory	2
	Cauliflower	0.110	w			Natl. Academy of Sciences	3
	Cauliflower	0.110	mn		5	Siciliano et al.	3
Brussels sprouts sub group	Brussels sprouts	0.080	mn	0.01-.27	30	French National Inventory	2
	Brussels sprouts	0.100	w			Natl. Academy of Sciences	3
	Brussels sprouts	0.100	mn		7	Siciliano et al.	3
Carrots (cooked, raw)[h]	Carrot	0.150	mn	0.007-2.80	80	French National Inventory	2
	Carrots	0.080	w			Howe et al.	1
	Carrot	0.080	w			Natl. Academy of Sciences	3
	Carrot	0.120	mn			Pobel et al.	2
	Carrot	0.060	mn		8	Siciliano et al.	3
	Carrot	0.100	mn		5	Siciliano et al.	3
	Carrot	0.110	mn		18	Siciliano et al.	3
	Carrot	0.250	mn	<0.5	8	Thomson and Swallow	2
	Carrot	0.010	mn	<0.02	7	Von Collet	2
Corn	Corn	0.200	w			Natl. Academy of Sciences	3
	Corn	0.200	mn		3	Siciliano et al.	3
Potatoes	Potatoes	0.015	mp	0.02-0.05	20	Food Standards Agency	2
	Potatoes (white)	0.160	mn	0.01-1.2	116	French National Inventory	2
	Potatoes, non specific	0.060	w			Howe et al.	1
	Potato	0.060	mn	0-.26	127	Petersen and Stoltze	2
	Potatoes	0.110	mn			Pobel et al.	2
	Potatoes (whole, small)	0.080	mn		1	Siciliano et al.	3
	Potatoes (whole)	0.050	mn		2	Siciliano et al.	3
	Potatoes (sliced)	0.040	mn		1	Siciliano et al.	3
	Potatoes	0.250	mn	<0.5	8	Thomson and Swallow	2
Soy milk or soy yogurt	Soybean sprouts	0.090	mn		40	Chung et al.	5
Spinach or collard greens							
Spinach sub group	Spinach	1.000	mn	0.03-22.0	41	French National Inventory	2
	Lettuce	0.040	w			Natl. Academy of Sciences	3
	Spinach	0.250	w			Natl. Academy of Sciences	3
	Lettuce	0.040	w			Natl. Academy of Sciences	3
	Lettuce	0.013	mn			Petersen and Stoltze	2
	Spinach	0.450	mn		20	Petersen and Stoltze	2
	Spinach	1.100	mn		36	Petersen and Stoltze	2
	Lettuce	0.020	mn		304	Petersen and Stoltze	2
	Lettuce	0.680	mn			Pobel et al.	2
	Spinach	0.770	mn			Pobel et al.	2
	Spinach	0.070	mn		7	Siciliano et al.	3
	Lettuce	0.040	mn			Siciliano et al.	3
	Spinach	0.610	mn		4	Siciliano et al.	3
	Spinach	0.070	mn		1	Siciliano et al.	3
	Lettuce	0.250	mp	<0.5	18	Thomson and Swallow	2
	Spinach	0.250	mp	<0.5	8	Thomson and Swallow	2
	Spinach	0.380	mn	<0.02-1.96	6	Von Collet	2

	Lettuce	0.010	mn	<0.02	28	Von Collet	2
Collard greens sub group	Collard greens	0.170	mn		4	Siciliano et al.	3
	Mustard greens	0.160	mn		4	Siciliano et al.	3
	Turnip greens	0.440	mn		3	Siciliano et al.	3
	Collard greens	0.020	mn		2	Siciliano et al.	3
	Turnip greens	0.020	mn		2	Siciliano et al.	3
	Mustard greens	0.030	mn		2	Siciliano et al.	3
Squash	Squash (acorn)	0.040	mn			Siciliano et al.	3
	Squash (butternut)	0.040	mn			Siciliano et al.	3
	Squash (zucchini)	0.060	mn			Siciliano et al.	3
	Squash	0.090	mn		2	Siciliano et al.	3
	Zucchini	0.100	mn		4	Siciliano et al.	3
String Beans	Green beans	1.260	mn			Pobel et al.	2
	Green beans	0.090	mn		4	Siciliano et al.	3
	Green beans	0.020	mn		4	Siciliano et al.	3
Tofu, tempeh or soy burgers	Soybean sprouts	0.090	mn		40	Chung et al.	5
Yams or sweet potatoes	Sweet potatoes	0.070	w			Natl. Academy of Sciences	3
**Fats, Oils, Nuts, and Sweets**							
Butter	Butter and cream	0.000	mn			Pobel et al.	2
Cake or donut	Cake	0.130	mn			Pobel et al.	2
Candy without chocolate	Sugar, sweets	0.000	mn			Pobel et al.	2
Chocolate	Sugar, chocolate, sweets	0.000	mn			Pobel et al.	2
Cookies[d]	Sugar, butter, milk, eggs, flour	0.043	mn				
French fried potatoes	Potatoes (hash brown)	0.070	mn		2	Siciliano et al.	3
Margarine	Margarine	0.000	w			Howe et al.	1
Nuts	Nuts	0.035	mp	<0.04-0.05	20	Food Standards Agency	2
Oil and Vinegar dressing	Salad dressing	0.000	mn			Howe et al.	2
	Oil	0.000	mn			Pobel et al.	2
Peanut butter	Nuts	0.035	mp	<0.04-0.05	20	Food Standards Agency	2
Pie	Pie	0.110	mn			Howe et al.	1
Potato chips or corn chips	Potatoes (hash brown)	0.070	mn		2	Siciliano et al.	3

[a] Mean is either the average, weighted mean, midpoint, or half the detection limit when reported as less than the detection limit as listed under nitrite value type.

[b] mn=mean, mp=mid point, w = weighted mean

[c] Information provided when reported in the literature; nd=not detected; tr = trace amount

[d] recipe calculation

[e] item used in recipe calculation for salsa

[f] Beef, pork, lamb as a sandwich or mixed dish and beef, pork, lamb as a main dish are made up of the same values, only the serving size is different.

[g] Carrots raw or cooked are made up of the same values, only the serving size is different.

Reported nitrosamine content of items listed in the food database and reference information used to generate the summary estimates by category

NBDPS Food Item	Food Name	Mean[a] (µg/100g)	Total Sample Size[b]	Author	Rank
**Dairy Products**					
Cheese	Cheese	0.200		Howe et al.	1
	Cheese (blue)	0.050	14	Klein et al.	2
	Cheese (soft)	0.020		Klein et al.	2
	Cheese	0.050	12	Osterdahl	2
	Cream cheese	0.001		Pobel et al.	2
	Hard cheese	0.045		Pobel et al.	2
	Soft cheese	0.045		Pobel et al.	2
	Cheese	0.270	209	Spiegelhalder	2
	Cheese	0.034	8	Tricker et al.	2
Cottage or ricotta cheese	Cheese	0.200		Howe et al.	1
	Cheese (blue)	0.050	14	Klein et al.	2
	Cheese (soft)	0.020		Klein et al.	2
	Cheese	0.050	12	Osterdahl	2
	Cream cheese	0.001		Pobel et al.	2
	Hard cheese	0.045		Pobel et al.	2
	Soft cheese	0.045		Pobel et al.	2
	Cheese	0.270	209	Spiegelhalder	2
	Cheese	0.034	8	Tricker et al.	2
Ice cream[c]	Milk, sugar, eggs	0.043			
Skim or low fat milk	Milk	0.080	57	Havery et al.	1
	Milk	0.051		Pobel et al.	2
	Milk	0.045	9	Tricker et al.	2
	Milk	0.190	7	Libbey et al.	3
Whole milk	Milk	0.010	10	Lakritz and Pensabene	1
	Milk	0.045	9	Tricker et al.	2
Yogurt	Yogurt	0.001		Pobel et al.	2
**Fruit Products**					
Avocado or guacamole	Fresh fruit	0.000		Pobel et al.	2
	Fruit, fresh	0.005	24	Tricker et al.	2
	Fruit, products	0.005	8	Tricker et al.	2
Bananas	Fresh fruit	0.000		Pobel et al.	2
	Fruit, fresh	0.005	24	Tricker et al.	2
	Fruit, products	0.005	8	Tricker et al.	2
Cantaloupe	Fresh fruit	0.000		Pobel et al.	2
	Fruit, fresh	0.005	24	Tricker et al.	2
	Fruit, products	0.005	8	Tricker et al.	2
Fresh apples or pears	Fresh fruit	0.000		Pobel et al.	2
	Fruit, fresh	0.005	24	Tricker et al.	2
	Fruit, products	0.005	8	Tricker et al.	2

Fruit drinks	Fruit juices	0.000		Howe et al.	1
Orange juice	Oranges	0.000		Howe et al.	1
Oranges	Oranges	0.000		Howe et al.	1
Other fruits	Fresh fruit	0.000		Pobel et al.	2
	Fruit, fresh	0.005	24	Tricker et al.	2
	Fruit, products	0.005	8	Tricker et al.	2
Peaches, apricots, plums, or nectarines	Fresh fruit	0.000		Pobel et al.	2
	Fruit, fresh	0.005	24	Tricker et al.	2
	Fruit, products	0.005	8	Tricker et al.	2
Raw chile peppers	Green beans	0.000		Pobel et al.	2
Salsa (fruit or tomato)[c]	Tomato, peppers, onions	0.000			
Onion[d]	Green beans	0.000		Pobel et al.	2
Tomatoes or tomato juice	Tomatoes	0.000		Howe et al.	1
	Tomato	0.000		Pobel et al.	2
**Grain Products**					
Biscuits, scones, croissants, muffins	Biscuits	0.015	3	Tricker et al.	2
Cereal	Cereal, cooked	0.000		Howe et al.	1
	Cereal products	0.050	11	Osterdahl	2
	Cereal products	0.018	10	Tricker et al.	2
Dark bread	Wheat flour	0.000		Pobel et al.	2
Rice or pasta	Pasta	0.000		Howe et al.	1
	Rice, non specific	0.000		Howe et al.	1
	Pasta, rice	0.000		Pobel et al.	2
Tortilla	Bread (non specific)	0.000		Howe et al.	1
	White bread	0.000		Pobel et al.	2
	Bread (non specific)	0.015	3	Tricker et al.	2
White bread	Bread (non specific)	0.000		Howe et al.	1
	White bread	0.000		Pobel et al.	2
	Bread (non specific)	0.015	3	Tricker et al.	2
**Meat and Bean Products**					
Bacon	Bacon	2.100	18	Canas et al.	1
	Bacon	0.500		Howe et al.	1
	Bacon	2.107	39	Vecchio et al.	1
	Bacon	0.140	5	Klein et al.	2
	Bacon	0.930	68	Osterdahl	2
	Bacon	0.111	4	Tricker et al.	2
Beans or lentils	Peas	0.000		Pobel et al.	2
Beans, refried	Peas	0.000		Pobel et al.	2
Beef, pork, lamb[e]					
Pork sub group	Pork	0.000		Howe et al.	1
	Pork	2.825	17	Sen et al. 1988	1
	Ham	0.020	4	Klein et al.	2
	Ham	0.010	4	Klein et al.	2
	Ham (non smoked)	0.100	9	Klein et al.	2
	Pork	0.500	21	Osterdahl	2

	Pork	0.350	18	Osterdahl	2
	Ham	0.040		Pobel et al.	2
Beef sub group	Corned beef	0.000		Howe et al.	1
Meat sub group	Meat products	0.100	23	Osterdahl	2
	Meat products	0.090	14	Osterdahl	2
	Red meat	0.000		Pobel et al.	2
	Smoked meat	0.035		Pobel et al.	2
Chicken livers	Liver, beef	0.000		Howe et al.	1
	Pâté (campagne)	0.040	2	Klein et al.	2
Chicken or turkey	Poultry	0.011		Pobel et al.	2
	Poultry	0.128	3	Tricker et al.	2
Eggs	Egg	0.000		Howe et al.	1
	Egg	0.000		Pobel et al.	2
Fish	Fish, other	0.000		Howe et al.	1
	Fish, pickled	3.200		Howe et al.	1
	Fish, salted	3.200		Howe et al.	1
	Fish, smoked	2.590		Howe et al.	1
	Fish	0.020	53	Cornee	2
	Fish	2.590		Cornee	2
	Fish	0.105		Key et al.	2
	Baltic herring	0.250	5	Osterdahl	2
	Baltic herring (fermented)	0.030	1	Osterdahl	2
	Fish	0.150	61	Osterdahl	2
	Salmon (pickled)	0.080	8	Osterdahl	2
	Fish (pickled)	0.110		Pedersen and Meyland	2
	Fresh fish	0.022		Pobel et al.	2
	Smoked fish	2.590		Pobel et al.	2
	Fish, fresh	0.300	8	Tricker et al.	2
	Fish, smoked	0.143	4	Tricker et al.	2
	Fish, tinned	0.210	5	Tricker et al.	2
Hamburger	Meat products	0.100	23	Osterdahl	2
	Meat products	0.090	14	Osterdahl	2
	Red meat	0.000		Pobel et al.	2
	Smoked meat	0.035		Pobel et al.	2
Hot dogs	Sausage (beef and pork)	0.060		Howe et al.	1
	Saucisson	0.660	8	Klein et al.	2
	Cured meat (sausage)	0.215		Pobel et al.	2
	Sausage	0.092	14	Tricker et al.	2
Liver	Liver, beef	0.000		Howe et al.	1
	Pâté (campagne)	0.040	2	Klein et al.	2
Organ meats and tongue	Offals	0.000		Pobel et al.	2
	Offals	0.097	3	Tricker et al.	2
Peas or lima beans	Peas	0.000		Pobel et al.	2
Processed meats					
Sausage sub group	Sausage (beef and pork)	0.060		Howe et al.	1
	Saucisson	0.660	8	Klein et al.	2

	Cured meat (sausage)	0.215		Pobel et al.	2
	Sausage	0.092	14	Tricker et al.	2
Salami sub group	Salami	0.660	8	Klein et al.	2
	Salami	1.985	10	Yamamoto et al.	5
Pâté sub group	Pâté (campagne)	0.040	2	Klein et al.	2
Preserved meat sub group	Preserved meat	0.035		Pobel et al.	2
**Vegetable Products**					
Broccoli	Green beans	0.000		Pobel et al.	2
Cabbage, cauliflower, or Brussels sprouts	Cabbage	0.000		Howe et al.	1
	Cabbage	0.000		Pobel et al.	2
	Cabbage	0.000		Siciliano et al.	3
	Kale	0.000		Siciliano et al.	3
	Kale	0.000		Siciliano et al.	3
	Sauerkraut	0.000		Siciliano et al.	3
Carrots (cooked, raw)[f]	Carrots	0.000		Howe et al.	1
	Carrot	0.000		Pobel et al.	2
Corn	Green beans	0.000		Pobel et al.	2
Potatoes	Potatoes, non specific	0.000		Howe et al.	1
	Potatoes	0.000		Pobel et al.	2
Soy milk or soy yogurt	Soya sauce	0.000	4	Osterdahl	2
Spinach or collard greens	Lettuce	0.000		Pobel et al.	2
	Spinach	0.000		Pobel et al.	2
Squash	Green beans	0.000		Pobel et al.	2
String beans	Green beans	0.000		Pobel et al.	2
Tofu, tempeh or soy burgers	Soya sauce	0.000	4	Osterdahl	2
Yams or sweet potatoes	Potatoes, non specific	0.000		Howe et al.	1
	Potatoes	0.000		Pobel et al.	2
**Fats, Oils, Nuts, and Sweets**					
Butter	Butter and cream	0.000		Pobel et al.	2
Cake or donut	Cake	0.000		Pobel et al.	2
Candy without chocolate	Sugar, sweets	0.034		Pobel et al.	2
Chocolate	Chocolate bar	0.030	15	Osterdahl	2
	Chocolate beverage powders	0.030	2	Osterdahl	2
	Cocoa	0.070	12	Osterdahl	2
	Sugar, chocolate, sweets	0.034		Pobel et al.	2
Cookies[c]	Sugar, butter, milk, eggs, flour	0.007			
French fried potatoes	Potatoes, non specific	0.000		Howe et al.	1
	Potatoes	0.000		Pobel et al.	2
	Potatoes (hash brown)	0.000		Siciliano et al.	3
Margarine	Margarine	0.000		Howe et al.	1
Nuts	Salad dressing	0.000		Howe et al.	2
	Oil	0.000		Pobel et al.	2
Oil and vinegar dressing	Salad dressing	0.000		Howe et al.	2
	Oil	0.000		Pobel et al.	2

Peanut butter	Salad dressing	0.000		Howe et al.	2
	Oil	0.000		Pobel et al.	2
Pie	Pie	0.000		Howe et al.	1
Potato chips or corn chips	Potatoes, non specific	0.000		Howe et al.	1
	Potatoes	0.000		Pobel et al.	2
**Alcoholic Beverages**					
Beer	Beer	0.280		Howe et al.	1
	Beer	0.044		Cornee	2
	Beer	0.030	258	Osterdahl	2
	Beer	0.028		Pobel et al.	2
	Beer	0.310	190	Spiegelhalder	2
	Beer	0.165	99	Spiegelhalder	2
	Beer	0.024	39	Tricker et al.	2
	Beer	0.248	15	Sen et al. 1980	3
Wine	Wine	0.015	180	Osterdahl	2
	Wine	0.015	27	Tricker et al.	2
	Wine	0.005	8	Sen et al. 1980	3
Liquor and mixed drinks	Liquor	0.100		Howe et al.	1
	Whisky	0.130	45	Osterdahl	2
	Spirits	0.020	39	Osterdahl	2
	Spirits	0.180		Pobel et al.	2
	Spirits	0.015	48	Tricker et al.	2
Malt beverages	Imported malt	0.355	30	Osterdahl	2
	Domestic malt	0.065	48	Osterdahl	2
	Malt beverages	0.030	39	Osterdahl	2
	Malt beverages	0.073	12	Tricker et al.	2
	Whiskey	0.040	1	Sen et al. 1980	3

[a] Displayed is the sum of all types of nitrosamines reported. Most estimates reported in literature did not permit range for total nitrosamines.

[b] Information provided when reported in the literature

[c] Recipe calculation

[d] Item used in recipe calculation for salsa

[e] Beef, pork, lamb as a sandwich or mixed dish and beef, pork, lamb as a main dish are made up of the same values, only the serving size is different.

[f] Carrots raw or cooked are made up of the same values, only the serving size is different.
